# London Lancet

**Published:** 1861-08

**Authors:** 


The London *Lancet*, for July, is as heavily freighted as usual, with the rich fruits of the minds of Great Britain's most eminent men. The first dozen pages are devoted to clinical matter,—Holmes Coote on Chronic Diseases of Bones and Joints, Chambers' first Drill for Auscultation (which we shall give in our next), and Gairdners' Observations in the Royal

Infirmary, Edinburgh. Tilt, Skey, Wilson, and others, furnish interesting original papers; and the remainder of the table of contents presents a perfect *embarras des richesse* to one seeking to select its most valuable features. The Decennial Index, from '51 to '60, accompanies this number.

James Herald, 24 Ann street, New York, is the American publisher; Norris & Hyde, Madison street, Chicago, Illinois agents.

